# Neurostimulation of the Cholinergic Anti-Inflammatory Pathway Ameliorates Disease in Rat Collagen-Induced Arthritis

**DOI:** 10.1371/journal.pone.0104530

**Published:** 2014-08-11

**Authors:** Yaakov A. Levine, Frieda A. Koopman, Michael Faltys, April Caravaca, Alison Bendele, Ralph Zitnik, Margriet J. Vervoordeldonk, Paul Peter Tak

**Affiliations:** 1 SetPoint Medical Corporation, Valencia, California, United States of America; 2 Division of Clinical Immunology and Rheumatology, Academic Medical Center/University of Amsterdam, Amsterdam, the Netherlands; 3 Bolder BioPATH, Inc., Boulder, Colorado, United States of America; 4 Arthrogen BV, Amsterdam, the Netherlands; 5 GlaxoSmithKline, Stevenage, United Kingdom; 6 University of Cambridge, Cambridge, United Kingdom; Georgia Regents University, United States of America

## Abstract

**Introduction:**

The inflammatory reflex is a physiological mechanism through which the nervous system maintains immunologic homeostasis by modulating innate and adaptive immunity. We postulated that the reflex might be harnessed therapeutically to reduce pathological levels of inflammation in rheumatoid arthritis by activating its prototypical efferent arm, termed the cholinergic anti-inflammatory pathway. To explore this, we determined whether electrical neurostimulation of the cholinergic anti-inflammatory pathway reduced disease severity in the collagen-induced arthritis model.

**Methods:**

Rats implanted with vagus nerve cuff electrodes had collagen-induced arthritis induced and were followed for 15 days. Animals underwent active or sham electrical stimulation once daily from day 9 through the conclusion of the study. Joint swelling, histology, and levels of cytokines and bone metabolism mediators were assessed.

**Results:**

Compared with sham treatment, active neurostimulation of the cholinergic anti-inflammatory pathway resulted in a 52% reduction in ankle diameter (p = 0.02), a 57% reduction in ankle diameter (area under curve; p = 0.02) and 46% reduction overall histological arthritis score (p = 0.01) with significant improvements in inflammation, pannus formation, cartilage destruction, and bone erosion (p = 0.02), accompanied by numerical reductions in systemic cytokine levels, not reaching statistical significance. Bone erosion improvement was associated with a decrease in serum levels of receptor activator of NF-κB ligand (RANKL) from 132±13 to 6±2 pg/mL (mean±SEM, p = 0.01).

**Conclusions:**

The severity of collagen-induced arthritis is reduced by neurostimulation of the cholinergic anti-inflammatory pathway delivered using an implanted electrical vagus nerve stimulation cuff electrode, and supports the rationale for testing this approach in human inflammatory disorders.

## Introduction

Rheumatoid arthritis (RA) is a chronic inflammatory disease, characterized by swollen and tender joints and progressive destruction of cartilage and bone, leading to significant morbidity and increased mortality. RA is currently treated with targeted biological and oral drugs, which have greatly improved disease outcome, however there remains a need for additional and better treatment options. The recently discovered inflammatory reflex, an evolutionarily conserved reflex neural circuit that modulates innate and adaptive immunity [1], has enabled the development of a new therapeutic paradigm.

In the inflammatory reflex, mediators of inflammation are sensed by the peripheral and central nervous system and are reflexively down regulated via the prototypical efferent arm, termed the “cholinergic anti-inflammatory pathway” (CAP). CAP signaling is initiated in brainstem nuclei of the vagus nerve, and continues through the efferent vagus to synapses in the celiac and other peripheral ganglia. From the celiac ganglion, functional signals continue through the splenic nerve to synapse-like junctions with a subset of splenic CD4^+^CD44^high^CD62^low^ acetylcholine-producing T cells which signal to adjacent splenic macrophages bearing the alpha 7 nicotinic acetylcholine receptor (α7nAChR) [2], [3]. Macrophages respond to T cell cholinergic signaling by reducing cytokine release in response to Toll Like Receptor (TLR)-mediated local proinflammatory signals, thereby completing the reflex loop. We have postulated that although this reflex normally serves to maintain an appropriate level of response to infection and injury, it might be harnessed therapeutically to reduce pathological levels of inflammation by activating the CAP using electrical neurostimulation of the vagus nerve (NCAP) [1], [4].

We have previously proposed the use of implantable medical devices to deliver NCAP therapeutically for RA [5]. Studies using the rodent collagen-induced arthritis (CIA) model have demonstrated reduced disease in response to small molecule α7nAChR agonists and worsened disease after vagotomy or targeted disruption of the α7nAChR gene [6], [7]. The current experiments show for the first time that the severity of CIA can be reduced by NCAP delivered using an implanted electrical vagus nerve stimulation (VNS) cuff electrode, analogous to those now in clinical use for drug-resistant epilepsy [8].

## Methods

### Ethics statement

The study was approved by Bolder BioPATH's Institutional Animal Care and Use Committee. Animal care including room, cage and equipment sanitation conformed to the guidelines cited in the Guide for the Care and Use of Laboratory Animals [9] and the applicable standard operating procedures of Bolder BioPATH.

### Animals

Female Lewis rats (177–199 grams, 45 days old) were obtained from Charles River Laboratories, Inc., (Wilmington, MA, USA) and the study was conducted at Bolder BioPATH (Boulder, CO, USA). Animals were housed in a laboratory environment with temperatures ranging between 67–76°F and relative humidity between 30–70%. Automatic timers provided 12 hours of light and 12 hours of dark. Animals were allowed access *ad libitum* to Harlan Teklad Rodent Chow (Denver, CO, USA) and fresh municipal tap water.

### Study design

Rats were divided into four groups (**Table 1**): 1) lead surgically implanted, CIA induced, active NCAP-treated (CIA/NCAP (n = 9)), 2) lead surgically implanted, CIA induced, sham-NCAP treated (CIA/Sham NCAP (n = 12)), 3) lead surgically implanted, no CIA induced, active NCAP-treated (Control/NCAP (n = 4)), 4) no lead implanted, no CIA induced (Control/No implant (n = 4)).

**Table 1:** Treatment Groups.

	Lead Implanted	CIA Induction	Stimulation Delivery
CIA/NCAP	**+**	Active	Active
CIA/Sham NCAP	**+**	Active	Sham
Control/NCAP	**+**	Sham	Active
Control/No implant	**-**	Sham	N/A

### Surgical implantation of vagus nerve stimulation electrodes

**Figure 1:**
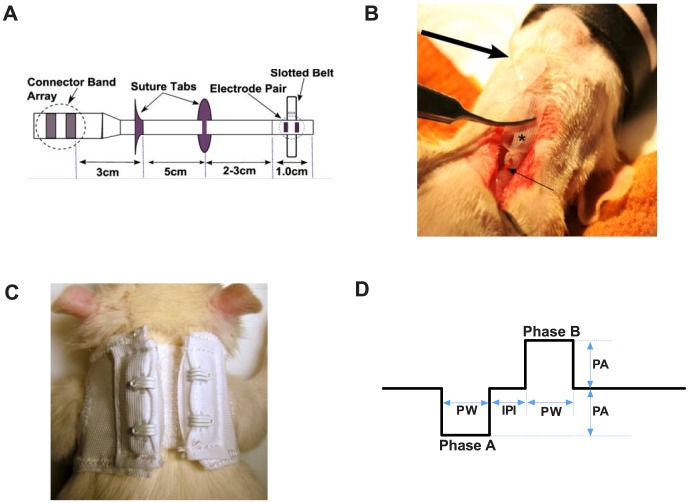
NCAP Delivery System. (A) Schematic drawing of the percutaneous vagus nerve cuff electrode (B) Surgical implantation of the lead in the neck of a supine animal is shown. The slotted belt (asterisk) is wound around the entire carotid sheath (small arrow) containing the vagus nerve, bringing the electrode pair in close apposition to and in proper orientation on the nerve, allowing effective induction of vagus depolarization. The distal portion of the lead (large arrow) is tunneled subcutaneously to the back, and externalized between the scapulae. (C) Following surgical healing, the distal end of the lead is externalized and protected under a jacket with a Velcro closure. The distal lead is intermittently connected using alligator clips to an external pulse generator for daily active or sham NCAP delivery. (D) The waveform of one cycle of the charge-balanced biphasic pulse delivered by the pulse generator is illustrated schematically. Rats were stimulated once daily for 60 seconds from study day 9 to 15 with a pulse waveform amplitude (PA) of 3 mA, pulsewidth (PW) of 200 microseconds, pulse frequency of 10 Hz, and a 50 microsecond inter-pulse interval (IPI).

The rats in the three implanted groups (CIA/NCAP, CIA/Sham, Control/NCAP) were anesthetized with isoflurane and secured in supine position. A ventral midline cervical incision was made between the mandible and sternum, and the mandibular salivary glands were bluntly separated and retracted laterally. The left carotid sheath was isolated between the sternomastoid and sternohyoid muscles. A custom-built bipolar cuff electrode (Evergreen, Minneapolis, MN, USA) (**Figure 1A**) with a silastic coated platinum wire lead, was secured about the outside of the entire carotid sheath containing the vagus nerve (**Figure 1B**) and gently tightened manually with a belt. The lead was anchored to the left sternomastoid, providing slack in the wire to the cuff to prevent displacement. The rat was then turned to a prone position and a dorsal midline incision was made between the scapulae. A subcutaneous tunnel was created and the distal end of the electrode pulled through and tunneled beneath the skin of the back. The lead was secured to the trapezius, the distal end was left in place subcutaneously, and the surgical wound closed. After recovery, the implanted rats were fitted with Velcro jackets used to protect the electrodes and were acclimated to the jackets for approximately 6 days (**Figure 1C**). Acclimation and recovery were complete when the rate of body weight increase paralleled that of non-implanted rats (Control/No implant group). After acclimation, the distal ends of electrodes were exteriorized from beneath the skin on day 0, while the animals were under anesthesia for CIA induction injections. The leads remained externalized and under the protective jacket through day 16, and were connected intermittently by alligator clips to an external pulse generator for delivery of daily active or sham stimulation.

### Neurostimulation

A custom-built external pulse generator (SetPoint Medical, Valencia, CA, USA) delivered charge-balanced biphasic pulses (**Figure 1D**) using a bipolar current source capacitively isolated with >1uF ceramic capacitors on both electrode outputs. Conscious rats in active stimulation groups (CIA/NCAP, Control/NCAP) were stimulated once daily from day 9 to day 15 for 60 seconds with a pulse waveform amplitude of 3 mA, 200 microsecond pulsewidth, a 50 microsecond inter-pulse interval, with a pulse frequency of 10 Hz. Rats in the CIA/Sham NCAP group were manipulated similarly, but received sham stimulation (output current of 0 mA). Visual confirmation of successful electrical stimulation was provided through the pulse generator software and noted following each stimulation.

### Induction of arthritis and assessment of disease activity

CIA/NCAP and CIA/Sham NCAP groups received injections of Freund's Incomplete Adjuvant (Difco, Detroit, MI) containing 2 mg/mL bovine type II collagen (Elastin Products, Owensville, Missouri) at the base of the tail and 2 sites on the back on days 0 and 6. Clinically apparent disease onset typically occurs at day 10 [10], [11]. The Control/NCAP and Control/No Implant groups were sham-injected with saline.

Caliper measurements of ankle diameter (Digitrix II micrometer; Fowler & NSK, Newton, MA, USA) were taken daily from day 9 until end of study at day 16. Disease activity was quantified using the group mean ankle joint diameter on each study day. Percent reduction in ankle diameter relative to arthritis controls (CIA/Sham NCAP) was calculated. Total area under the curve (AUC) was calculated for ankle diameter over time using the formula: 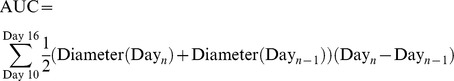



### Histology

Hind paws were transected at the level of the medial and lateral malleolus, and fixed in formalin. Decalcified joints were cut in half longitudinally (ankles) or in the frontal plane (knees), processed, sectioned, and stained with Toluidine Blue. A board certified veterinary pathologist who was blinded to treatment groups examined tissues microscopically. Joints were histologically scored (0 = normal; 5 = severe disease) for inflammation, cartilage damage, pannus formation, and bone resorption according to criteria shown in **Table 2**.

**Table 2:** Histopathology Scoring Criteria.*

Score	Inflammation	Pannus Formation	Cartilage Damage	Bone Resorption
0	None Present	None Present	None Present	None Present
1	Minimal infiltration in periarticular tissue	Minimal infiltration of pannus in cartilage and subchondral bone	Minimal to mild loss of Toluidine Blue staining no chondrocyte loss or collagen disruption	Small areas of resorption, rare osteoclasts
2	Mild infiltration	Mild infiltration (<1/4 of tibia at edges)	Mild loss of Toluidine Blue staining focal chondrocyte loss and collagen damage	More numerous areas of resorption, osteoclasts <1/4 of tibia at edges is resorbed
3	Moderate infiltration with moderate edema	Moderate infiltration (1/4 to 1/3 of tibia affected, smaller tarsals affected)	Moderate loss of Toluidine Blue staining with mid zone chondrocyte loss and collagen damage	Obvious resorption of medullary trabecular and cortical bone w/o full thickness defects in cortex, loss of some medullary trabeculae
4	Marked infiltration with marked edema	Marked infiltration (1/2-3/4 of tibia affected, destruction of smaller tarsals)	Marked loss of Toluidine Blue staining with deep zone chondrocyte loss and collagen damage	Full thickness defects in cortical bone, marked loss of medullary bone, numerous osteoclasts, 1/2–3/4 of tibia affected, destruction of smaller tarsals
5	Severe infiltration with severe edema	Severe infiltration (>3/4 of tibia affected, severe distortion of overall architecture)	Severe diffuse loss of Toluidine Blue staining with multifocal severe (to tide mark) chondrocyte loss and/or collagen disruption	Full thickness defects in cortical bone, marked loss of medullary bone, numerous osteoclasts, >3/4 of tibia affected

*Adapted from: Bendele, A., et al., *Effects of interleukin-1 receptor antagonist in a slow-release hylan vehicle on rat type II collagen arthritis.* Pharm Res, 1998. **15**(10): p. 1557–61.

### Assessment of mediators of inflammation and bone metabolism

Animals were deeply anesthetized for terminal blood draw prior to being euthanized. Cytokines (Interleukin (IL)-1α, IL-1β, IL-2, IL-6, Interferon (IFN)-γ and tumor necrosis factor (TNF)), receptor activator of nuclear factor kappa-B ligand (RANKL), osteoprotegerin (OPG), osteoclast-derived tartrate-resistant acid phosphatase form 5b (TRAP-5b), C-terminal peptide fragment of type I collagen (CTX-1), osteocalcin, and procollagen type 1 N-terminal propeptide (P1NP) were assessed in serum from the day 16 terminal bleed. Analysis of cytokines was performed by multi-analyte array (QAR-CYT-3 Quantibody array; Raybiotech, Norcross, GA, USA). RANKL and OPG were measured using ELISA (Cusabio, Wuhan, P.R. China). TRAP-5b and CTX-1 were measured using solid phase immunofixed enzyme activity assay and competitive binding enzyme-immunoassay (Immuno Diagnostic Systems, Bolden, UK), respectively. Osteocalcin and P1NP were measured using ELISA (Immuno Diagnostic Systems, Bolden, UK). All assays were performed according to manufacturer's instructions. Samples that fell below the lower limit of detection (LLD) were assigned a value of the LLD for that assay.

### Statistical Analysis

Differences between treatment group means in ankle diameter and AUC ankle diameter were analyzed by ANOVA with Holm-Sidak correction for multiple comparisons. Differences between CIA treatment group means in histological score, cytokine levels, and bone metabolism markers were analyzed with a Student's t-test. Significance for all tests was set at p<0.05. All statistical tests were performed using Prism 6.0 software (GraphPad software, San Diego, CA, USA).

## Results

### NCAP Reduces Clinical Signs of Joint Inflammation

**Figure 2:**
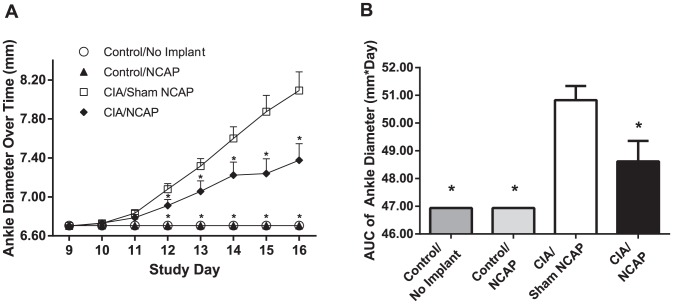
NCAP Reduces Clinical Signs of Joint Inflammation. Rats were placed into four treatment groups, three of which received vagus nerve lead implants, with a fourth group of unimplanted controls. The three implanted groups had CIA induction or sham CIA induction, and active versus sham electrical neurostimulation on study days 9–15 as outlined in Table 1 (Control/No Implant n = 4; Control/NCAP n = 4; CIA/NCAP n = 9; CIA/Sham NCAP n = 12). Ankle diameter over time is shown as mean+SE (A), and AUC of ankle diameter over Day 9–16 is shown as mean+SE (B), *p≤0.05 ANOVA versus CIA/Sham NCAP.

CIA/NCAP, CIA/Sham NCAP, and Control/Implant groups were treated with active or sham electrical stimulation of the vagus nerve, once daily for 60 seconds. Treatment began at day 9, three days after the second CIA immunization and one day prior to the typical onset of clinical disease in this model, and continued through day 15, with euthanasia on day 16. Beginning at day 12, ankle swelling, as assessed by caliper measurement, was significantly reduced in the group receiving active NCAP treatment (CIA/NCAP) when compared to sham NCAP-treated controls (CIA/Sham NCAP). There was no effect of the implant alone (Control/Implant) on ankle diameter in the absence of CIA induction (**Figure 2A**). At day 16 the mean difference in ankle diameter between CIA/NCAP and CIA/Sham NCAP was 0.72±0.26 mm, corresponding to a 52% reduction in swelling. The AUC of the ankle diameter from day 9 to 16 was also significantly reduced in the CIA/NCAP compared with CIA/Sham NCAP groups (48.62±0.74 mm*Day versus 50.83±0.51 mm*Day, respectively, a reduction of 57%, p = 0.02, **Figure 2B**).

### NCAP Reduces Histological Measures of Joint Damage

**Figure 3:**
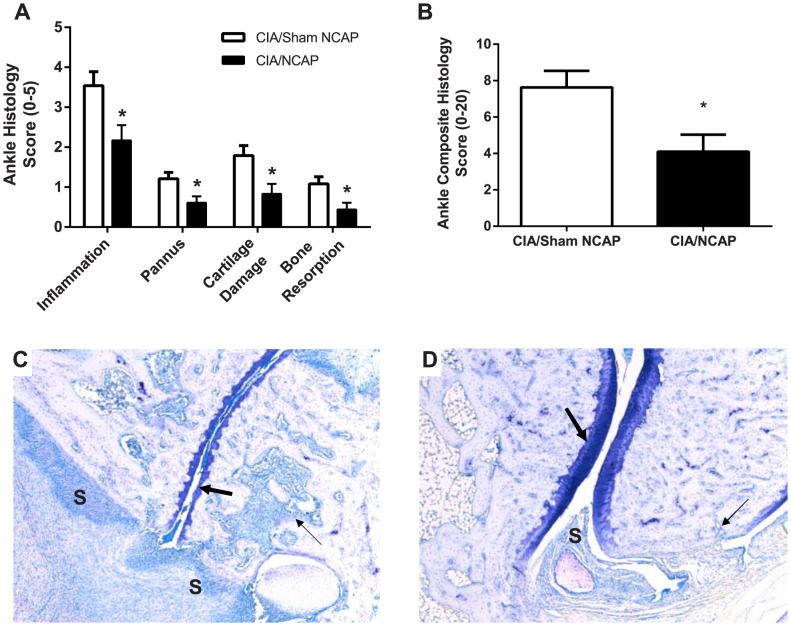
NCAP Reduces Histological Measures of Joint Damage. (A–B) Ankle joints were harvested on study day 16, stained with Toluidine Blue, and scored on a scale of 0-5 for inflammation, pannus formation, cartilage damage and bone resorption (A), with a composite summated score of 0–20 (B). Data are shown as mean+SE score. *p≤0.05 t-test versus CIA/Sham NCAP. (C–D) Representative 50X photomicrographs of ankle joints are shown which have the approximate mean summed score as that of the entire treatment group. Ankle from CIA/Sham NCAP group (C) demonstrates marked inflammation and synovitis (**S**) and mild cartilage damage (large arrow) and bone resorption (small arrow). Ankle from CIA/NCAP group (D) demonstrates mild inflammation and synovitis (S) and minimal cartilage damage (large arrow) and minimal bone resorption (small arrow).

At day 16, joints were assessed histologically in a blinded manner for presence of inflammation, pannus formation, cartilage damage, and bone resorption using a semi-quantitative scoring system (**Table 2**). Each of these parameters was graded between 0–5, and a composite score summed score was calculated. A 46% reduction in the composite score was observed (4.1±0.9 versus 7.6±0.9 in CIA/NCAP and CIA/Sham NCAP, respectively, p = 0.01, **Figure 3A**), and there were significant reductions in inflammation (2.2±0.4 versus 3.5±0.4), pannus formation (0.6±0.2 versus 1.2±0.2), cartilage damage (0.8±0.2 versus 1.8±0.2), and bone resorption (0.4±0.2 versus 1.1±0.2), mean±SE, p = 0.01, 0.01, 0.01, 0.02 respectively (**Figure 3B**). No effects on joint histology were seen in the animals implanted in the absence of CIA induction (Control/No Implant and Control/Implant composite scores were 0.0 and 0.0, respectively). **Figures 3C–D** illustrate typical histological appearance of joint inflammation and damage in CIA/Sham NCAP group and reductions induced by active treatment in the CIA/NCAP group. Similar effects were seen in the histological scores of the knee joint (data not shown).

### NCAP Effects on Systemic Cytokine Production

**Figure 4:**
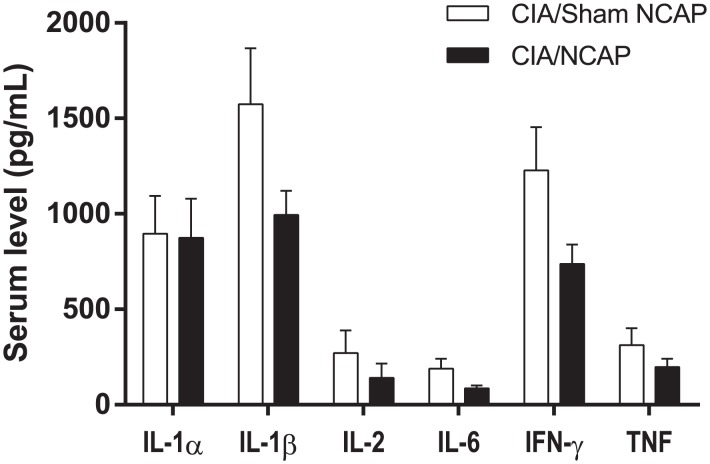
NCAP Effect on Circulating Cytokines. Study day 16 serum was assayed for IL-1α, IL-1β, IL-2, IL-6, IFN-γ and TNF. Data are shown as mean+SE level, t-test p = NS for all individual cytokine comparisons between CIA/NCAP and CIA/Sham NCAP.

To assess whether NCAP-induced improvements in ankle swelling and histological joint inflammation were accompanied by reductions in systemic pro-inflammatory cytokine production, IL-1α, IL-1β, IL-2, IL-6, IFN-γ and TNF were measured in serum at day 16. When CIA/NCAP and CIA/Sham groups were compared there was no between-group difference in IL-1α. In contrast, IL-1β, IL-2, IL-6, IFN-γ and TNF were reduced by 37%, 48%, 54%, 40%, and 37%, however none of these reductions in individual mediator levels reached statistical significance (**Figure 4**).

### NCAP Effects on Mediators of Bone Metabolism

Juxta-articular, peri-articular, and systemic bone loss occur in both RA and in the rat CIA model, driven primarily by direct increases in the pro-resorptive mediator RANKL and secondarily by increases in IL-1 and TNF, which can increase resorption both directly, as well as secondarily by inducing RANKL production (Redlich, 2012). To determine whether the observed reductions in histological bone erosions were accompanied by changes in systemic mediators of bone metabolism, RANKL and its decoy receptor/antagonist OPG, as well as bone resorption markers TRAP-5b and CTX-1, and bone formation markers osteocalcin and P1NP were measured at day 16.

**Figure 5:**
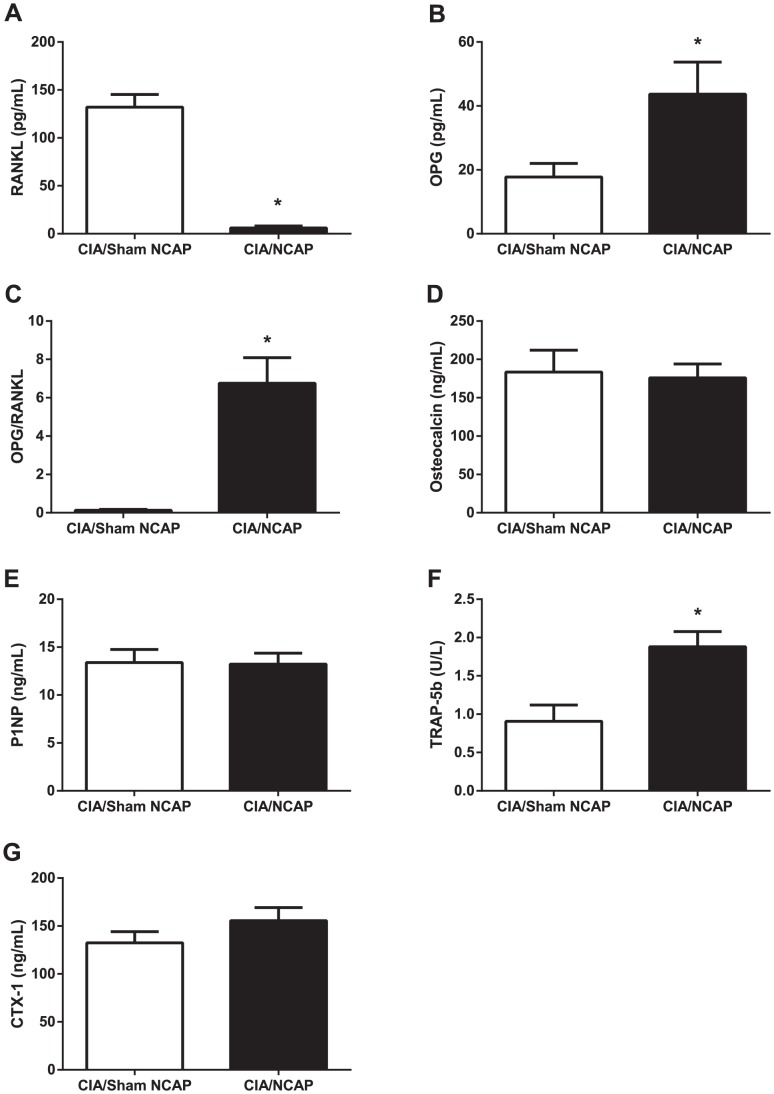
NCAP Effect on Mediators of Bone Metabolism. Study day 16 serum was assayed for RANKL (A), and OPG (B), and the ratio of OPG/RANKL group means calculated (C). Data are shown as mean+SE level, *p≤0.05, t-test versus CIA/Sham NCAP. Osteocalcin (D), P1NP (E), TRAP-5b (F) and CTX-1 (G) data are shown as mean+SE level, *p≤0.05 t-test versus CIA/Sham NCAP.

Serum levels of RANKL were reduced from 132±13 to 6±2 picograms/mL, OPG increased from 18±4 to 44±10 picograms/mL, and OPG/RANKL ratio increased from 0.1±0.1 to 6.8±1.3 in the CIA/Sham versus CIA/NCAP groups, respectively (p = 0.01, 0.02, 0.01), indicating a marked NCAP-induced reduction in propensity toward bone resorption at day 16 (**Figure 5A–C**). There were no treatment-induced changes in the bone formation markers osteocalcin and P1NP (**Figure 5D–E**). Interestingly, despite the greatly reduced levels of RANKL, and the markedly elevated OPG/RANK ligand ratio, which would be expected to drive diminished bone resorption, systemic markers typically indicative of resorption were either not changed (CTX-1), or were increased (TRAP-5b) in the CIA/NCAP group, compared to CIA/Sham NCAP (**Figure 5F–G**).

## Discussion

Activation of the CAP by neurostimulation or pharmacologic means reduces inflammation through a variety of mechanisms. As noted above, the release of chemokines, cytokines, and other mediators of inflammation from monocytes and macrophages during their response to local and systemic pro-inflammatory signals is inhibited. In addition, CAP activation reduces neutrophil CD11b expression [12], and diminishes neutrophil trafficking to sites of inflammation in skin, joint and gut [12]–[14]. Further, subdiaphragmatic vagotomy increases and pharmacologic CAP agonists decrease *in vitro* T cell proliferation and secretion of IFN-γ, TNF, and IL-6 [15]. *In vitro* culture of naïve T cells with nicotine enhances the effect of cell activation-induced expression of FoxP3, and nicotine treatment markedly increases the influx of CD4^+^CD25^+^FoxP3^+^ T regulatory cells (Treg) into the gut in rodent oxalazone-induced colitis [16]. In rodent hapten-induced colitis, disease severity is worsened by subdiaphragmatic vagotomy, correlated with reductions in FoxP3^+^ Tregs. Over time the proinflammatory effect of vagotomy wanes, accompanied by recovery of Treg numbers [17], [18]. Finally, effects on B cells have also recently been demonstrated. In response to VNS or cholinergic agonists, splenic marginal zone B cells exhibit reduced trafficking to the splenic red pulp and peri-follicular areas during their maturation process. This migratory arrest is driven by changes in CD11b, and is associated with reduced secretion of antibodies [19]. These inhibitory effects on mononuclear cell mediator release, neutrophil trafficking, and T and B cell function may be contributing to the improvements in CIA disease measures we have observed.

Our use of electrical neurostimulation to elicit CAP activation in CIA is supported by several prior studies. Because of the critical role of the α7nAChR and its expression within the fibroblast-like synoviocytes of inflamed human synovium [20], [21] we previously studied the course of CIA in mice with targeted disruption of the receptor gene [7]. When compared to wild type littermates, knockout animals had a faster onset, greater incidence, and worsened severity of disease; increased radiographic evidence of bone destruction, increased histological joint inflammation, elevated *in vitro* release of Th1 cytokines from cultured splenocytes and increased systemic levels of Monocyte Chemotactic Peptide (MCP)-1 and TNF. Conversely, the course and severity of murine CIA was ameliorated by systemic treatment with the selective α7nAChR agonist AR-R17779 [6]. Rats that underwent indirect vagus nerve stimulation by surgically suspending the nerve against the sternocleidomastoid muscle prior to CIA induction had significant improvements in paw volume, clinical arthritis score, radiographic assessment of bone erosions, histological evidence of erosions and inflammation, and reduced serum TNF levels [22]. The present study reveals that successful treatment delivery can be accomplished using a simple surgically implanted cuffed nerve electrode system analogous to those used in many other clinical applications in humans.

The “dosing paradigm” chosen for the study (pulse amplitude, frequency and pulse width stimulation parameters) was guided by prior experience in rodent endotoxemia and other acute injury models [1], generally recognized conditions necessary for efficient vagus nerve depolarization [23], [24], and published clinical experience in vagus nerve stimulation for epilepsy and other conditions [25]. One remarkable aspect of NCAP delivery is that relatively brief periods of nerve stimulation result in a prolonged biological effect. Mice given a 60 second stimulation had reductions in LPS-induced systemic TNF production for up to 48 hours [26]. Similar prolonged protective effects have been seen in preliminary studies in a normal canine model (Levine, unpublished data), and in a rat model of indomethacin-induced enteropathy [27]. We chose the 1-minute daily dosing paradigm for this study based on these data. The mechanism of this prolonged effect is not yet well understood. Interestingly, human peripheral blood mononuclear cells differentiated to a macrophage-like phenotype by *in vitro* culture with GM-CSF released reduced levels of TNF for up to 48 hours after a 60 minute period of culture in acetylcholine [26], indicating that the prolonged effect may be due to changes in the function of monocytes and other hematopoietic cells rather than in the nervous system.

NCAP treatment reduced bone erosions, in association with marked reductions in systemic RANKL, the major regulator of osteoclast maturation, function, and survival [28], and concomitant increases in the RANKL antagonist OPG. IL-1 and TNF are well known inducers of RANKL, and antagonism of these cytokines reduces bone loss in CIA [29], [30]. Direct RANKL antagonism causes reduction in bone loss in the absence of effects on local or systemic inflammation, in both CIA [30] and in human RA [31]. We did not observe effects of NCAP on the bone formation markers P1NP and osteocalcin, consistent with the more limited effect of anti-inflammatory treatments on systemic measures of osteoblast function [32]. However we also did not observe a reduction in systemic CTX-1, a marker of bone resorption, and saw an increase in the resorption marker TRAP-5b. Previous detailed studies of the kinetics of TRAP-5b in rat CIA have shown up to 3-fold day to day differences in circulating levels during the first 14 days after disease onset [33]. It is therefore possible that the absence of reductions in these resorption markers, which is typically observed, may be related to the relatively short duration of the experiment and single time point of sampling. However, the mechanism of effects in this neurostimulation treatment model may be more complex. It has recently been demonstrated that the skeleton is functionally innervated by vagus neurons in mice, and that subdiaphragmatic vagotomy or targeted disruption of the α2 N-acetylcholine receptor (α2NAChR) results in reduced bone mass on micro-CT, and *in vitro* exposure to nicotine induces apoptosis of osteoclasts which bear the α2NAChR [34]. The authors postulate a CNS-controlled bone regulatory system analogous to the inflammatory reflex. Given this complexity, elucidation of the precise mechanism responsible for NCAP-induced RANKL inhibition and reduction in articular bone loss we observed will require further studies of marker kinetics, cytometry, and histology in both CIA and ovariectomy models.

The study has some limitations. Although there was good rationale for the stimulation delivery parameters chosen, the human diseases and animal models used to inform these decisions are different in pathogenesis than CIA, and it is possible that with higher stimulation levels, or more frequent delivery, even greater efficacy might have been observed. The lead system used required extended maintenance of percutaneously-externalized leads, which were not possible to keep sterile, and were not physically robust enough to last more than approximately 4–5 weeks without breakage, requiring us to limit the planned length of the overall experiment to approximately 3 weeks. Given the constant movement of the leads and the lack of sterility, we cannot exclude the possibility that low grade subcutaneous inflammation may have been partially responsible for the large degree of between-animal variability seen in serum cytokine levels in both treatment and control groups, perhaps limiting our ability to detect statistically significant treatment-related differences. In addition, the potential activation of pressoreceptors or musculature in the artery by the electrodes wrapped around the carotid-bundle may have contributed indeterminate effects. Availability of more advanced lead prototypes that are small enough to be placed directly on the vagus nerve, and not around the entire carotid sheath, and a fully implantable pulse generator, similar to those used in humans, will solve these problems in the future. Finally, as noted, the short duration of the experiment, and availability of only an end-of-study blood sample limited the ability to fully understand the kinetics of the bone turnover markers.

## Conclusions

Prior studies of electrical neurostimulation were mainly done in models driven by tissue injury or alterations in innate immune response. This is the first study to demonstrate that electrical neurostimulation using an implanted vagus nerve stimulation cuff electrode can activate the cholinergic anti-inflammatory pathway and significantly reduce activity in a widely accepted and utilized preclinical autoimmune disease model. This study supports the rationale for testing this approach in human immune-mediated inflammatory disorders, such as the recently started pilot studies in inflammatory post-operative ileus (NCT 01572155), Crohn's disease (NCT 01569503), and RA (NCT 01552941), and research on the development of bioelectronic medicines [35]. Success in these and subsequent studies may eventually lead to novel alternative therapeutic options for patients suffering from RA and other inflammatory disorders.
